# Hemorrhagic shock from post-traumatic rupture of microcystic splenic lymphangioma: A case report and review of the literature

**DOI:** 10.1016/j.ijscr.2020.09.045

**Published:** 2020-09-10

**Authors:** Giuseppe Evola, Giovanni Mazzone, Antonino Corsaro, Giovanna Brancato, Francesco Roberto Evola, Guido Basile

**Affiliations:** a General and Emergency Surgery Department, Garibaldi Hospital, Piazza Santa Maria di Gesù 5, 95100, Catania, Italy; b First Department of Surgery, Azienda Ospedaliero-Universitaria “Policlinico-V. Emanuele-San Marco”, P.O. San Marco, Viale C.A. Ciampi, 95121, Catania, Italy; c Department of General Surgery and Medical-Surgical Specialties, University of Catania, Via S. Sofia 78, 95123, Catania, Italy; d Department of Orthopedic and Traumatology, Cannizzaro Hospital, Via Messina 829, 95126, Catania, Italy

**Keywords:** Cystic lymphangioma, Spleen, Hemorrhagic shock, Splenectomy, Case report

## Abstract

**Introduction:**

Lymphangioma is an abnormal proliferation of lymphatic vessels. Isolated splenic lymphangioma (SL) is rare; in the last 30 years only 22 cases were reported in the literature. Diagnosis is difficult by the absence of typical symptoms and signs. SL is often asymptomatic and occasionally detected through radiological exams or, after the onset of a life-threatening complication, at surgery or pathological examination. Surgery represents the treatment of choice.

**Presentation of case:**

A 22-year-old Caucasian male was admitted to the Emergency Department after a car accident, complaining of sudden onset, severe, left upper quadrant abdominal pain. Abdominal examination revealed mild abdominal distention and a severe abdominal pain on superficial and deep palpation of left upper abdominal quadrant with obvious muscle guarding and rebound tenderness. Abdominal computed tomography scan showed splenic rupture with hemoperitoneum. Laboratory tests reported severe anemia. The patient received blood transfusions and was taken emergently to the operating room for exploratory laparotomy, evacuation of hemoperitoneum and splenectomy. The postoperative course was uneventful, the patient was discharged on the 7th post-operative day. Diagnosis of microcystic SL was made at pathological examination.

**Discussion:**

Post-traumatic splenic rupture may be favored by the presence of a unknown and asymptomatic isolated SL. Although different types of treatment of SL are reported in the literature, splenectomy represents the treatment of choice to avoid complications.

**Conclusion:**

Isolated SL is difficult to diagnose and suspect because of lack of pathognomonic symptoms and specific diagnostic signs. It can be diagnosed after the onset of a life-threatening complication. Splenectomy is the preferable definitive treatment.

## Highlights

• Lymphangioma is considered an abnormal proliferation of lymphatic vessels.
• Preoperative diagnosis of isolated splenic lymphangioma is very difficult because it is rare and often asymptomatic.
• Undiagnosed microcystic splenic lymphangioma represents a risk factor for post-traumatic splenic rupture, even after minor abdominal trauma.
• Total splenectomy is considered the correct treatment to avoid complications.
• Definitive diagnosis of isolated splenic lymphangioma can be provided after a histopathological examination.

## 1 Introduction

Lymphangioma is considered an abnormal proliferation of lymphatic vessels that mainly develops in children and rarely in adults. It is debated whether it is a benign tumor or a congenital malformation of the lymphatic system [1]. Lymphangioma commonly involves the neck (75%) and axilla (20%) but can occur sporadically in the orbit, lung, bone, skin, mediastinum, retroperitoneum, omentum and in many internal organs (gastro-intestinal tract, adrenal gland, kidney, spleen, liver, pancreas) either as systemic disease (lymphangiomatosis syndrome), or as a single organ lesion [2]. Isolated splenic lymphangioma (SL) is extremely rare: the first case was described in 1885 by Frink and only 22 cases, between 1990 and 2020, were reported in the literature. Preoperative diagnosis is difficult because of its rarity and the absence of typical symptoms and signs, however aid of imaging studies can make it easier. The clinical manifestations of SL are usually related to the size of the spleen. Generally SL is asymptomatic and incidentally detected through imaging studies [2]. The differential diagnosis is broad, the treatment of choice is splenectomy to avoid serious complications and prognosis is good [1]. SL has a high risk of rupture even after minor abdominal trauma [3]. A case of a young patient with hemorrhagic shock caused by post-traumatic rupture of a unknown SL, is presented with review of the literature in accordance with the Surgical Case Reports (SCARE) criteria [4].

## 2 Presentation of case

**Fig. 1:**
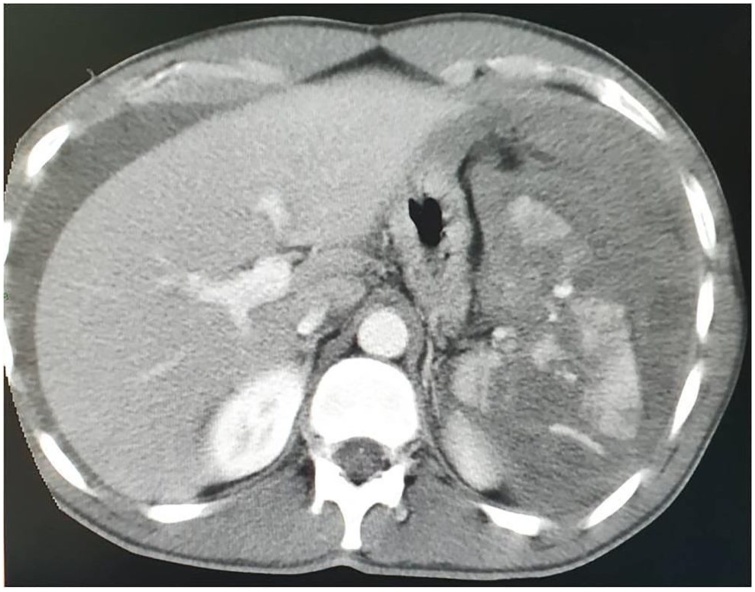
Abdominal CT scan showing splenic rupture with hemoperitoneum.

**Fig. 2:**
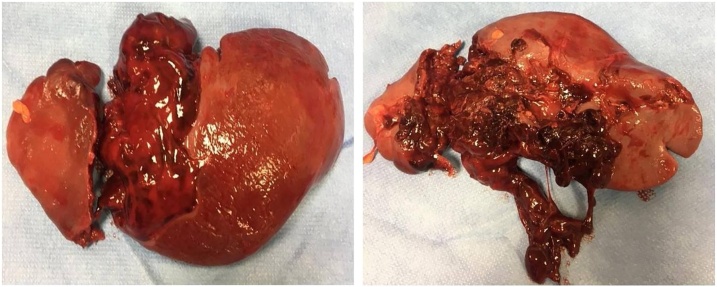
(a,b). Post-traumatic splenic rupture: operative finding.

**Fig. 3:**
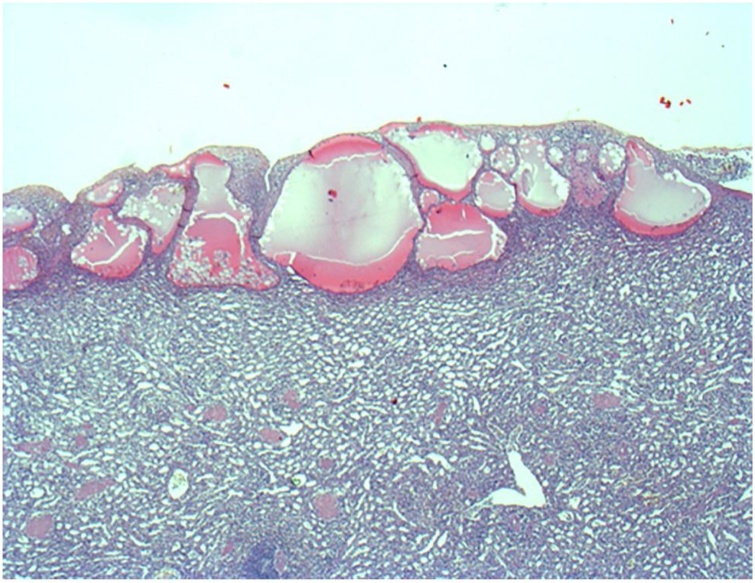
Photomicrograph section of the microcystic splenic lymphangioma (haematoxylin and eosin,orginal magnification ×20).

**Fig. 4:**
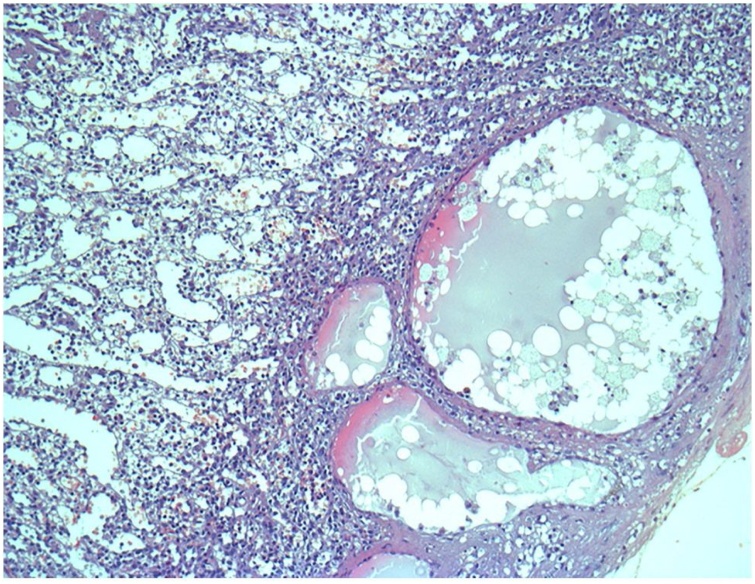
Photomicrograph section of the microcystic splenic lymphangioma (haematoxylin and eosin,orginal magnification ×100).

A 22-year-old Caucasian male was admitted to the Emergency Department after a car accident, complaining of sudden onset, severe, left upper quadrant abdominal pain. He was found to be hypotensive, pale and diaphoretic. Vital signs on admission were blood pressure 80/40 mm Hg, pulse 125 bpm, respiratory rate 26 per minute and no fever. The patient wasn’t taking any drugs or smoking and his past and familial medical histories were normal. Abdominal examination revealed mild abdominal distention and a severe abdominal pain on superficial and deep palpation of left upper abdominal quadrant with obvious muscle guarding and rebound tenderness. Treatment was initiated with crystalloid fluid bolus with appropriate improvement in blood pressure but persistent tachycardia. Thoracic and abdominal computed tomography (CT) scan showed splenic rupture with massive hemoperitoneum (Fig. 1). Laboratory tests reported anemia (hemoglobin 7.5 g/dl). Transfusion of packed red blood cells (PRBC) was initiated with transient hemodynamic response. The patient was treated by intravenous antibiotics and, after understanding the severity of his medical condition and accepting surgery, taken emergently to the operating room by experienced trauma surgeons for exploratory laparotomy, evacuation of hemoperitoneum and splenectomy. At laparotomy the spleen had several large areas of capsular tear (Fig. 2). In total the patient received four units of PRBC and two units of fresh frozen plasma perioperatively. The postoperative course was uneventful and laboratory tests reported a hemoglobin concentration of 11 g/dl. The patient received pneumococcal, meningococcal and Hemophilus vaccination and was discharged on the 7th postoperative day in a stable condition with the advice to undergo periodic vaccinations. The splenectomy specimen, sent for histopathology evaluation, measured 11.5 × 10 × 4 cm and weighed 180 g. On microscopic examination the splenic parenchyma showed many subcapsular cystically dilated lymphatic spaces lined by a single layer of flattened endothelial cells and filled with thick eosinophilic material without calcifications, atypia or malignancy (Fig. 3, Fig. 4). Diagnosis of microcystic SL was made. The patient after a follow-up of one year is asymptomatic.

## 3 Discussion

This clinical case describes the post-traumatic rupture of an asymptomatic microcystic SL causing hemorrhagic shock. Although no consensus has yet been reached on whether lymphangioma is a neoplasm or a hamartoma, most researches support the latter opinion. Its formation would be due to abnormal congenital development of lymphatic vessel or attributed to bleeding or inflammation in the lymphatic system, which causes obstruction and consequent lymphangiectasia. Based on the size of the dilated lymphatic channels, lymphangioma can be classified as capillary (supermicrocystic), cavernous (microcystic) or cystic (macrocystic) [2]; the microcystic type, discovered in our patient, isn’t the most common [5]. Lymphangiomas can be characterized pathologically by a flat epithelial endothelium and a wall containing alternatively lymphoid tissue, small lymphatic spaces, smooth muscle and foam cells [6]. SL can presents as a unique subcapsular cyst or multiple cystic lesions that can be intraparenchymal or sometimes replace the entire normal splenic parenchyma (splenic lymphangiomatosis). The typical histological aspect of SL after splenectomy shows cystic structures filled with eosinophilic, proteinaceous material and lined by flattened endothelial cells [7]. Most lymphangiomas are discovered in children, with 80–90% being diagnosed before the age of 2 [8]. Isolated SL is a very rare entity because of others organs are often involved as part of the so-called lymphangiomatosis syndrome. As isolated SL is often asymptomatic and represents an incidental radiological finding, especially if the lesion is small, preoperative diagnosis is very difficult; instead large cystic lesions can attain sufficient size to cause significant symptoms and signs which at the same time aren’t pathognomonic. In the case of a large SL, the symptomatology may include left upper quadrant pain with splenomegaly, nausea, vomiting, loss of appetite, shortness of breath, abdominal distention, further generalized symptoms secondary to compression of adjacent viscera or an acute abdomen, as in our patient, in case of complications like as rupture or infection [2]. The differential diagnosis is broad including hemangioma, splenic infarction, pseudocyst, epidermoid cyst, mesothelial cyst, parasitic or hydatid cysts, septic embolism, old hematomas, lymphoma or metastasis [9,10]. Preoperative diagnosis is difficult like as for other pathologies [11], depending on the non specificity of symptoms and signs; however it’s improved by medical imaging including abdominal ultrasound (US), abdominal CT scan and magnetic resonance imaging (MRI) [12]. The variability of lymphatic vessel caliber associated with some degree of fibrosis and calcification, provides a wide range of imaging aspects. Because lymphangiomas more commonly involve many organs at one time, the diagnostic evaluation must be extended to search for other sites affected by the disease [7]. US may reveal the presence of well-defined hypoechoic or anechoic cystic lesions with occasional internal septations or calcifications [7]. CT scan is the diagnostic technique of choice and shows multiple, low-density cysts without enhancement or with only slight enhancement of the thin septa, with or without mural calcification [13]. The cystic lesions are hypointense on T1-weighted MRI scans and hyperintense on T2-weighted MRI scans. MRI can facilitate the detection of solid areas into the lumen of cystic spaces suggesting malignant degeneration [3]. On angiography, these lesions are avascular [3]. PET-CT is rarely used for the diagnosis, excluding a malignant tumor where the other imaging techniques are inconclusive [5]. In our case abdominal CT scan, performed in emergency, wasn’t diagnostic for microcystic SL that was revealed only at pathological examination. Lymphangioma rarely regresses spontaneously and, if untreated, it persists and often expands (being locally invasive or even malignant), potentially damaging vital structures [14]. Complications of SL include rupture with hemorrhage and peritonitis, infection, abscess formation, pleural effusion, lung atelectasis, pneumonia, empyema, intestinal obstruction, diaphragmatic immobility, hypertension, tumor enlargement and a low risk of malignant transformation to lymphangiosarcoma [7,10]. Excessive growth of SL can lead to complications such as consumptive coagulopathy, hypersplenism and portal hypertension [15]. According to literature, in our case unknown microcystic SL represented a risk factor for post-traumatic splenic rupture, so diagnosis was made after detection of a complication. The treatment of SL depends on the lesion size and the presence of complications. Some investigators prefer conservative treatment in the case of small and asymptomatic lesions detected incidentally, reserving splenectomy for large, multiple or symptomatic lesions. Conservative management (aspiration, drainage or sclerosis) is associated with high risk of recurrence [5]. Medical treatment was described by Reinhardt using the alpha interferon in children with good tolerance, however the optimal dose and duration of this curative therapy is not known [14]. Although partial splenectomy has been used in case of limited disease, in order to avoid the overwhelming post-splenectomy infection [10], total splenectomy is the preferable definitive treatment to avoid complications. Laparoscopic splenectomy is emerging as the procedure of choice in patients with a normal to moderately enlarged spleen but it has multiples contraindications including portal hypertension with high hemorrhagic risk and massive splenomegaly. Accessory spleen must be removed because they can be concomitantly affected [16]. The prognosis of SL after splenectomy is favorable, but it has a high risk of splenic rupture and can recur after incomplete surgical resection (9.5% of patients) [5]. In our case open splenectomy was performed as life-saving treatment for the patient that had no complications after surgery.

## 4 Conclusion

SL is a rare malformation of the splenic lymphatic vessels, mostly seen in children and rarely in adults, often asymptomatic and incidentally detected through imaging studies. Because of the absence of pathognomonic symptoms and signs, preoperative diagnosis is difficult: in our case diagnosis was favoured by a life-threatening complication (splenic post-traumatic rupture) that represents a possible consequence of SL. Although different types of conservative treatment are reported in literature, total splenectomy represents the preferable definitive treatment to avoid possible lethal complications. Definitive diagnosis is achieved by histopathologic examination after splenectomy.

## Conflicts of interest

All the authors certify that there is no conflict of interest regarding the material discussed in the manuscript.

## Sources of funding

All the authors declare that this research didn’t receive any specific grant from funding agencies in the public, commercial, or not-for-profit sectors.

## Ethical approval

Ethical approval has been exempted by our institution because this is a case report and no new studies or new techniques were carried out.

## Consent

Written informed consent was obtained from the patient, for publication of this case report and accompanying images. A copy of the written consent is available for review by the Editor-in-Chief of this journal on request.

## Author contribution

Giuseppe Evola: Drafting the manuscript and literature research.

Giovanni Mazzone: Operated on the patient, literature research.

Antonino Corsaro: Operated on the patient, literature research

Giovanna Brancato: Drafting the manuscript and literature research.

Francesco Roberto Evola: Drafting the manuscript and literature research.

Guido Basile: Drafting the manuscript, literature research, revising the manuscript.

## Registration of research studies

This case report does not require registration as a research study.

## Guarantor

The guarantor for this case report is Giuseppe Evola.

## Provenance and peer review

Not commissioned, externally peer-reviewed.
